# Subcutaneous Methylnaltrexone for Opioid-Induced Constipation in Older Adults With Advanced Illness

**DOI:** 10.1093/geroni/igaf122.3377

**Published:** 2025-12-31

**Authors:** Julie Knight, Sri Nalamachu, Adam Laitman

**Affiliations:** Salix Pharmaceuticals, Bridgewater, New Jersey, United States; Analgesic Clinical Research, Blue Springs, Missouri, United States; Salix Pharmaceuticals, Bridgewater, New Jersey, United States

## Abstract

Opioid-induced constipation (OIC), affecting up to 81% of patients taking opioid analgesics, is caused by opioids binding to peripheral mu-opioid receptors in the gastrointestinal tract. Methylnaltrexone, a peripherally-acting mu-opioid receptor antagonist, reverses opioid-related constipating effects without affecting centrally-mediated analgesia. This analysis evaluated subcutaneous methylnaltrexone efficacy/safety in older adults (≥65 years) with advanced illness (eg, incurable cancer) and OIC. In 2 randomized, double-blind, placebo-controlled, phase 3 trials, patients received subcutaneous methylnaltrexone 0.15 or 0.30 mg/kg, or placebo. Primary endpoint (both trials) was percentage of patients with rescue medication-free bowel movement (RFBM) within 4 hours after first dose. Data were pooled and analyzed posthoc. Overall, 161 patients (49.1% female) were included. Significantly more patients aged ≥65 years treated with subcutaneous methylnaltrexone 0.15 (37/68 [54.4%]) or 0.30 mg/kg (16/31 [51.6%]) had an RFBM within 4 hours versus placebo (9/62 [14.5%]; both P < 0.001 vs placebo). When further stratified by age, an RFBM within 4 hours was reported in significantly more patients 65-74 years treated with methylnaltrexone 0.15 (13/31 [41.9%]) or 0.30 mg/kg (8/15 [53.3%]) versus placebo (3/23 [13.0%]; P ≤ 0.03 for both); for 75-84 years, methylnaltrexone 0.15 (17/25 [68.0%]) or 0.30 mg/kg (7/12 [58.3%]) versus placebo (3/21 [14.3%]; P < 0.001 and P = 0.02 versus placebo, respectively). For patients aged ≥85 years, significantly more patients had a RFBM within 4 hours in methylnaltrexone 0.15 mg/kg (7/12 [58.3%]) versus placebo (3/18 [16.7%]; P = 0.04) group. Subcutaneous methylnaltrexone was well tolerated. In conclusion, subcutaneous methylnaltrexone for OIC was efficacious, with a rapid onset of action, and well tolerated in older individuals.

